# Tuning Interlayer Molecular Weight in Electrodeposited Anion Exchange Membranes for Enhanced Reverse Electrodialysis Performance

**DOI:** 10.3390/polym18172104

**Published:** 2026-08-29

**Authors:** Aydın Cihanoğlu

**Affiliations:** 1 Department of Chemical Engineering, Ege University, Bornova, 35100 Izmir, Türkiye; aydin.cihanoglu@ege.edu.tr; Tel.: +90-232-616-7607; 2 Aliağa Vocational School, Refinery and Petrochemical Technology, Ege University, Aliaga, 35800 Izmir, Türkiye

**Keywords:** reverse electrodialysis, anion exchange membrane, layer-by-layer deposition, monovalent ion selectivity, uphill transport, anti-organic fouling

## Abstract

Renewable energy can be harvested from salinity gradients using reverse electrodialysis (RED); however, the open-circuit voltage and power output of this process can be significantly reduced by multivalent ions and natural organic matter found in natural waters. In this work, a tailor-made polyepichlorohydrin-based anion exchange membrane (AEM) surface was modified using an electrophoretic layer-by-layer (LbL) polyelectrolyte assembly. Negatively charged poly(styrene sulfonate) (PSS) and positively charged poly(ethyleneimine) (PEI) were employed to construct three-layer architectures in which PEI served as the interlayer. The results indicate that the molecular weight of the PEI interlayer strongly influences the surface composition and charge of the final AEMs. RED experiments performed in the presence of Na_2_SO_4_ revealed that AEMs incorporating the high-molecular-weight PEI exhibited enhanced apparent Cl^−^/SO_4_^2−^ selectivity and delivered an increased power density. Fouling tests using a real humic–fulvic acid mixture demonstrated that the hydrophilic PSS top layer effectively mitigated organic fouling and preserved RED performance. Furthermore, short-term stability testing provided a preliminary indication of the stability of the polyelectrolyte layers under short-term operating conditions. This study highlights the critical role of interlayer molecular weight in defining the surface chemistry, apparent ion selectivity, and antifouling behavior of LbL-modified tailor-made AEMs, providing important design guidelines for improving RED performance in realistic feedwaters.

## 1. Introduction

Meeting global energy demand through fossil fuels is becoming increasingly unsustainable, driving an urgent need for clean, reliable, and scalable alternatives. Although conventional renewable sources such as solar, wind, hydropower, wave, tidal, biomass, and geothermal energy play essential roles in the global energy portfolio, many of these technologies remain constrained by geographical, seasonal, or climatic variability [1,2,3,4,5,6,7]. In this context, salinity gradient energy (SGE) has attracted growing attention as a complementary renewable option due to its independence of external conditions, and it generates no solid, liquid, or gaseous waste [8,9,10,11,12]. Its theoretical potential, estimated at 1.4–2.6 TW globally based on Gibbs free energy calculations, corresponds to nearly 20% of current energy demand and could substantially reduce CO_2_, CH_4_, and N_2_O emissions if fully utilized [13].

Among existing SGE technologies, RED is considered the most promising due to its straightforward operation, high theoretical power density, and ability to convert natural salinity differences directly into electrical energy [14,15,16]. A RED stack consists of alternating anion and cation exchange membranes (AEMs and CEMs) placed between two flowing salt solutions of differing salinity. Ion transport driven by the salinity gradient creates a Nernst potential, which is harvested through redox reactions at the electrodes [10,12].

Despite extensive research, a major disconnect remains between laboratory studies typically conducted with pure NaCl solutions and real feedwaters such as seawater, brackish water, or estuarine mixtures [14,15,16]. Natural waters inevitably contain divalent ions (e.g., SO_4_^2−^, Mg^2+^, Ca^2+^) and NOM, including humic and fulvic acids. Even at low concentrations, these species significantly impair the performance of RED. Divalent ions increase ohmic resistance, reduce permselectivity, and undergo “uphill transport,” which diminishes open-circuit voltage (OCV) and overall power output. NOM, on the other hand, strongly interacts with positively charged AEM surfaces, neutralizing fixed charges and increasing membrane resistance, ultimately leading to severe power losses. Therefore, developing AEMs that can simultaneously achieve high monovalent/divalent ion selectivity and robust antifouling performance is critical for real-world RED applications [17].

Surface modification has emerged as one of the most effective strategies to address these challenges. Separating monovalent from divalent anions relies predominantly on size exclusion and electrostatic repulsion, whereas antifouling performance is governed by surface hydrophilicity and charge characteristics [10]. Among surface-tailoring techniques, LbL polyelectrolyte assembly is desirable due to its simplicity, versatility, and ability to form highly controlled, electrostatically cross-linked multilayers under mild conditions [18]. Recent advances indicate that appropriate selection of polyelectrolytes, deposition conditions (static or electric-field-assisted), and terminating-layer charge can significantly enhance ion selectivity and organic fouling resistance [18]. Despite these developments, most reported LbL-modified AEMs have been evaluated in electrodialysis (ED) rather than in RED, and systematic studies examining their impact on energy harvesting remain limited [19,20,21]. There are very few studies on this issue. For instance, Güler et al. [22] modified the surface of commercially available, low monovalent ion-selective AEMs through copolymerization to enhance their selectivity and reduce fouling for RED applications. While the desired results were not achieved with monovalent ion selectivity, the high negative charge of the modified AEMs imparted hydrophilic characteristics to the surface, resulting in a fouling-resistant structure. Chen’s research group modified the surfaces of commercial AEMs with 7.5 layers of polyelectrolytes using the LbL deposition under static conditions [23]. The results showed that the modified AEMs had increased Cl^−^/SO_4_^2−^ selectivity and organic fouling resistance, and that more energy was generated from the RED system compared to the bare AEM.

Furthermore, while several studies have explored the effect of layer number, polyelectrolyte type, ionic strength, and pH on LbL performance [18,24], the influence of interlayer molecular weight on RED-relevant properties has not been previously investigated. Given that polyelectrolyte molecular weight affects chain conformation, charge density distribution, and film morphology, it may play a decisive role in determining the final membrane’s surface chemistry, hydrophilicity, and ion selectivity.

In this work, a tailor-made PECH-based AEM was synthesized via solvent evaporation and subsequently modified with negatively charged PSS and positively charged PEI using electrophoretic LbL deposition. Furthermore, for the first time in the literature, the impact of interlayer molecular weights (25 kDa and 750 kDa) was examined on their effect on membrane surface properties, apparent Cl^−^/SO_4_^2−^ selectivity, antifouling behavior, and RED performance. The number of layers was intentionally limited to 1.5 bilayers to minimize additional mass transfer resistance. Membranes were characterized using contact angle measurements, zeta potential, SEM, and XPS analyses. RED experiments were conducted with monovalent and divalent ion mixtures at various flow velocities to evaluate uphill transport effects and net power density (P_Net_). Fouling resistance was assessed using a real mixture of humic and fulvic acids. This study provides the first systematic assessment of how interlayer molecular weight influences the structure–property–performance relationships of LbL-modified tailor-made AEMs in RED. The findings offer new insights for designing high-performance, fouling-resistant, and monovalent-selective membranes for practical salinity-gradient energy harvesting.

## 2. Materials and Methods

### 2.1. Materials

Polyepichlorohydrin (PECH, 37 wt.% chlorine) and polyacrylonitrile (PAN) were used as the active and inert polymers, respectively, for fabricating the tailor-made anion exchange membranes (AEMs). PECH was supplied by Osaka Soda Co., and PAN by Mitsubishi Chemical, Osaka, Japan. 1,4-Diazabicyclo[2.2.2]octane (DABCO, ≥99%, Sigma-Aldrich, St. Louis, MO, USA) served as both a crosslinker and amine reagent for PECH. Dimethyl sulfoxide (DMSO, Merck, Darmstadt, Germany) was used as the solvent for all polymer solutions.

For surface modification, poly(styrene sulfonate) (PSS, 70 kDa) and poly(ethyleneimine) (PEI, 25 kDa and 750 kDa) (PEI 25 kDa: Branched PEI with an average Mw ~25,000 Da (determined by Light Scattering), corresponding to an average number-average molecular weight Mn of ~10,000 Da by GPC; PEI 750 kDa: Branched PEI with an average Mw ~750,000 Da (determined by Light Scattering), corresponding to an average Mn of ~60,000 Da by GPC) were obtained from Sigma-Aldrich. Sodium chloride (NaCl) and sodium sulfate (Na_2_SO_4_) were purchased from Merck and used to prepare feed, electrode, and polyelectrolyte solutions. Potassium ferricyanide (K_3_Fe(CN)_6_) and potassium ferrocyanide (K_4_Fe(CN)_6_), both analytical grade (Merck), were used to prepare electrode rinse solutions in the RED system. A commercial humic–fulvic acid mixture (0.01 wt.%) (Ant Humix Liquid, Yem-Miks Company, Izmir, Türkiye) was used in the fouling experiments.

### 2.2. Membrane Preparation

Tailor-made PECH-based bare AEMs were synthesized using the solvent evaporation method [25,26,27]. Before dissolution, PECH granules were dried at 80 °C for 30 min to remove residual moisture. The dried PECH was then dissolved in DMSO at 80 °C under agitation (600 rpm) for 3 h. Separately, DABCO was dissolved in a small amount of DMSO at 25 °C and slowly added to the PECH solution. The mixture was stirred at 80 °C for an additional 6 h to promote crosslinking. PAN was then dissolved in DMSO at 25 °C and added to the PECH-DABCO reaction mixture. The final casting solution, containing 10 wt.% total polymers (PECH + PAN) with a fixed mass ratio of 0.33 (m_PECH_/m_PAN_) and molar ratio of 1.0 (n_DABCO_:n_CH2Cl_), was stirred at 25 °C for 18 h to form a homogeneous gel [28]. The viscous casting solution was spread onto a clean glass plate and adjusted to a wet-film thickness of 1000 µm. The film was dried at 80 °C for 6 h, cooled to room temperature, and then detached by immersing the glass plate in 0.5 M NaCl. The resulting membrane was stored in 0.5 M NaCl at 25 °C until use. This membrane is referred to as QPECH-PAN_R 1.0 [28].

### 2.3. Membrane Modification

LbL deposition was performed via an electrophoretic approach, in which charged polyelectrolytes were transported toward the membrane surface under an applied electric field. In this study, a constant current density of 7.5 mA cm^−2^ was applied during deposition, resulting in the electrophoretic migration of negatively charged PSS and positively charged PEI toward the oppositely charged membrane surface. Unlike conventional static LbL assembly, where polyelectrolyte adsorption is governed primarily by diffusion and electrostatic interactions, the present method involves electrophoretic transport, which enhances the deposition rate and promotes the formation of more uniform and compact multilayers. The applied current density controls the flux of charged species, thereby influencing layer thickness, density, and surface coverage. 

**Scheme 1:**
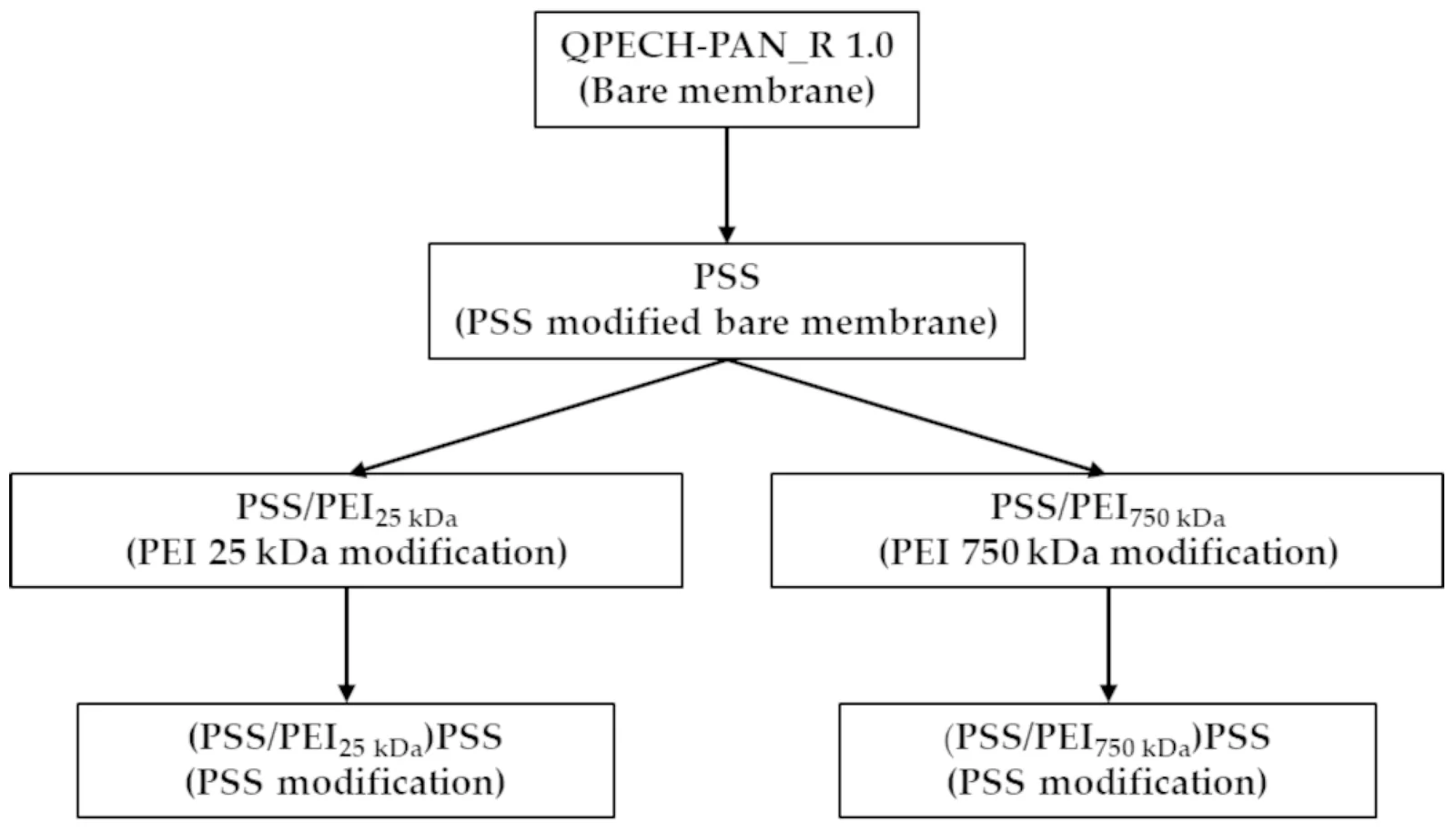
Modification steps of bare QPECH-PAN_R 1.0 and labels of the modified AEMs.

LbL deposition was performed with PSS in 0.5 M NaCl and PEI in 1.0 M NaCl to an active membrane area of 10 × 10 cm. The reason for preparing polyelectrolytes in the presence of salt is to reduce the repulsion force between the coated polyelectrolyte chains (screening effect) and to ensure that the surface is coated with more polyelectrolyte. The concentration of both polyelectrolytes was 1 g/L. During the modification, the molecular weight of the negatively charged PSS polyelectrolyte was kept constant at 70 kDa, and the molecular weight of the positively charged PEI polyelectrolyte was varied to 25 kDa and 750 kDa. The pH of the polyelectrolyte solutions was adjusted to 2.85. The aim is to increase the protonation of the positively charged amine groups in PEI during the modification process. This increases the electrical attraction between the positive and negative polyelectrolytes. The bare AEM has a positive charge due to the quaternary amine groups it contains; therefore, the modification of bare AEM surfaces with polyelectrolytes was first initiated with the negatively charged PSS. The first layer was modified for 30 min. Then, the PSS-coated membrane surface was modified with positively charged PEI for 15 min. As the top layer, positively charged membrane surfaces were modified with negatively charged PSS for 15 min. The reason is that the top layer is coated with a negatively charged polyelectrolyte to increase the surface’s hydrophilicity and resistance to organic contamination. Furthermore, the negative surface charge enables the removal of divalent anions through electrostatic repulsion. After each coating, membrane surfaces were washed with deionized water for 5 min to remove excess and weakly adsorbed polyelectrolytes. The modification was applied in 3 layers—1.5 bilayers—to minimize coating-induced mass transfer resistance on AEM surfaces. Scheme 1 shows the modification steps of the bare AEM and labels of the modified AEMs.

It should be noted that the system was operated under galvanostatic conditions; therefore, the electric field strength was not directly imposed but developed across the solution and membrane according to the system resistance. Under these conditions, the electric field can be qualitatively related to the applied current density through Ohm’s law, although its exact value depends on solution conductivity and electrode configuration. The use of electrophoretic LbL deposition enables more controlled and efficient formation of polyelectrolyte multilayers compared to diffusion-limited methods, particularly for dense and highly charged systems.

### 2.4. Characterization of Membranes

Water contact angle (WCA) measurements of the membranes were carried out using an Attension Optical Tensiometer. Before measurement, the AEMs, which had been kept in a 0.5 M NaCl solution, were thoroughly washed with pure water and dried in an oven at 35 °C overnight. The dried AEMs were then fixed to a glass surface using double-sided tape. A total of 5 µL of pure water was used for the measurements. Experiments were performed using six AEM coupons, and the results were determined by averaging repetitions.

The surface zeta potential of the AEMs was determined using a NanoPlus Micromeritics instrument at pH 6.5 (working pH) and in the presence of a 10^−2^ M NaCl electrolyte solution. Measurements were taken three times, and the results are given as the average of the measurements.

XPS analysis of the AEMs was performed by taking measurements from three different regions at an emission angle of 0°. Conducting XPS analysis at an emission angle of 0° indicates that information is collected from the surface at a maximum measurement depth of 10 nm.

The surface morphology of the AEMs was determined using scanning electron microscopy (FEI Quanta 250 FEG). Before SEM imaging, the dried AEMs were coated with gold using a magnetron sputter coating instrument to enhance their conductivity.

### 2.5. RED Performance Tests and Theoretical Background

The performance of the salinity gradient power generation of the AEMs was tested using the RED system (STT Products B.V., Schiedam, The Netherlands). Details about the RED system’s working principle, components, preparation of the electrode solution, and feed solutions can be found in the Supplementary Information (SI). The RED performance of the polyelectrolyte-modified AEMs was compared with that of the bare tailor-made AEM (QPECH-PAN_R 1.0). Fujifilm Type II CEM was used as a cation exchange membrane in the study, and relevant commercial CEM information is provided in the Supplementary Information (SI). Finally, the SI contains the calculations for the residence time and Reynolds number of the feed solutions, as well as the net power density (P_Net_) and pumping power density (P_Pump_) of the RED system. 

### 2.6. Stability and Organic Fouling Performance of AEMS

The stability of the AEMs was determined by testing the RED performance for 4 h under an optimum constant current density of 6 A/m^2^. The standard RED performance tests for AEMs involve comparing the power densities obtained when different currents are applied to the membranes. The constant current density value used in stability studies is the value at which the maximum power density is achieved. In this study, 0.513 M and 0.017 M NaCl solutions were used as the concentrate and dilute solutions, respectively, in the stability performance tests. The flow velocity of 0.52 cm/s, which produced the peak P_Net_ value among the tested parameters, was selected as the optimum solution flow velocity.

The fouling behavior of AEMs against organic pollutants was determined using a real mixture of humic and fulvic acids (Ant Humix Liquid, Yem-Miks Company). The AEMs in our study were kept statically immersed in a solution of 0.513 M NaCl + 20 ppm humic and fulvic acid (total volume 1 L) at 25 °C for 7 days. A commercial stock solution of humic–fulvic acid mixture at 0.01 wt.% (equivalent to 100 ppm) was utilized. The final working solution was prepared by diluting an appropriate volume of this commercial stock to a target concentration of 20 ppm in a 1 L volumetric flask. Then, any weakly attached organic acids on the AEMs’ surface were removed by washing the membranes with pure water. Following fouling, the RED performance of the fouled AEMs was measured using a pair of 0.513 M and 0.017 M NaCl solutions at a constant flow velocity of 0.52 cm/s at 25 °C. Additionally, the stability performance of fouled AEMs was also evaluated.

### 2.7. Statistics

An unpaired Student’s *t*-test was used to determine statistically significant differences between all of the data. The graphs use asterisks (*) to denote significance, which is defined in the figure captions. 

To ensure the reliability and reproducibility of the findings, all Reverse Electrodialysis (RED) and fouling performance experiments were conducted in triplicate using three distinct, independently prepared membrane samples (each with a 10 cm × 10 cm active area). The data points presented in the figures express the calculated arithmetic mean of these independent measurements. Due to the high reproducibility of the fabrication and testing processes, the standard deviations for all performance indicators were remarkably low, consistently falling within the order of magnitude of 10^−4^. Consequently, the resulting error bars are visually smaller than the data symbols/markers used in the plots and are thus enclosed within them.

## 3. Results and Discussion

### 3.1. Characterization of Membranes

**Figure 1:**
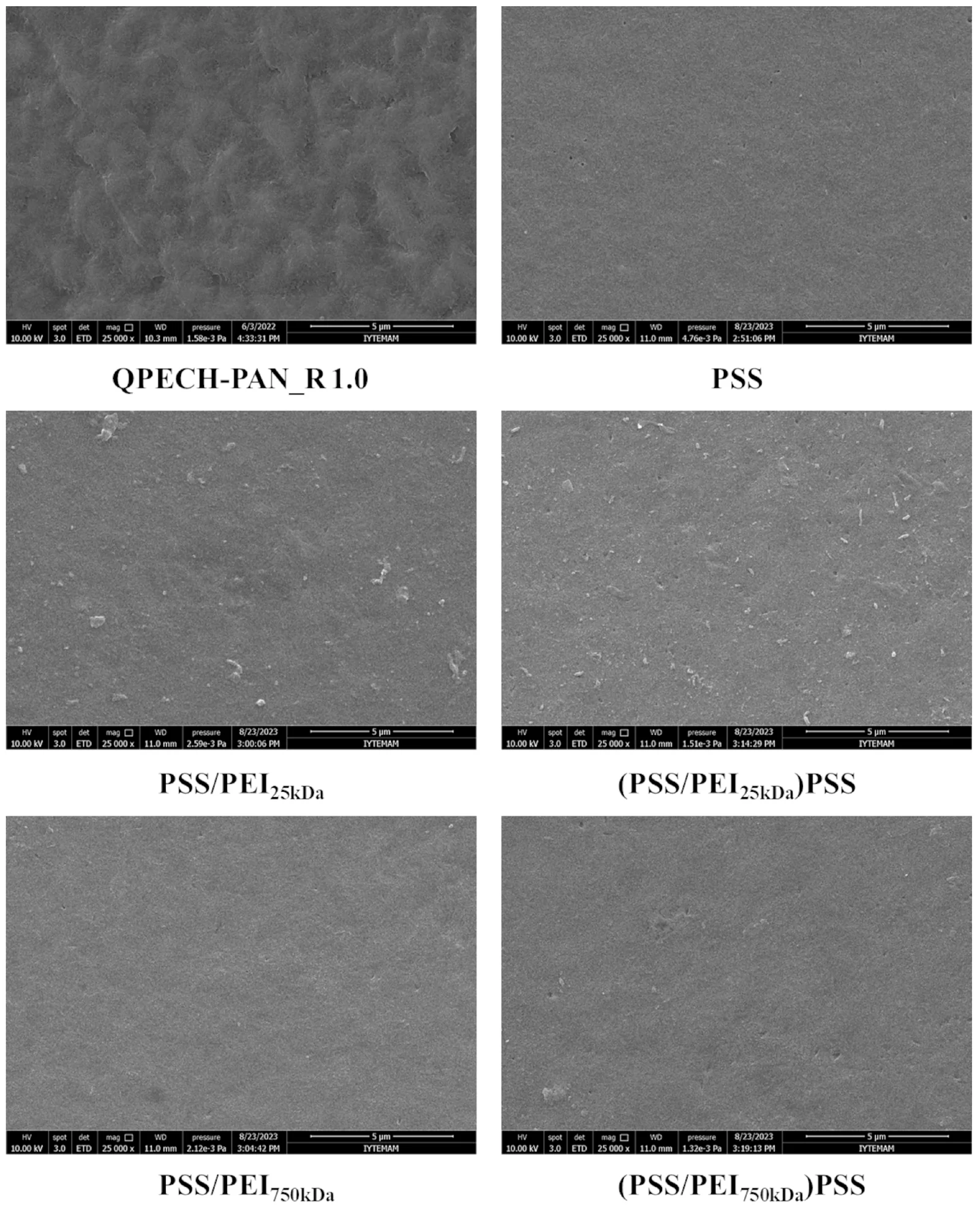
Surface SEM images of the bare and polyelectrolyte-modified AEMs. The performance data for the bare (QPECH-PAN_R 1.0) membrane used as a control group were adapted from our previous study [28] with proper copyright permission from the publisher.

Figure 1 presents the surface SEM images of the bare and polyelectrolyte-modified AEMs (25k magnification). The pristine QPECH-PAN_R 1.0 membrane exhibits a relatively rough surface with characteristic ridge-and-valley morphology. After LbL deposition, the surfaces become visibly smoother, indicating the formation of uniform polyelectrolyte layers. This smoothing effect is consistent with previous studies in which electric-field-assisted deposition produced dense, conformal coatings on AEMs [19]. The images qualitatively confirm successful modification of polyelectrolytes.

**Figure 2:**
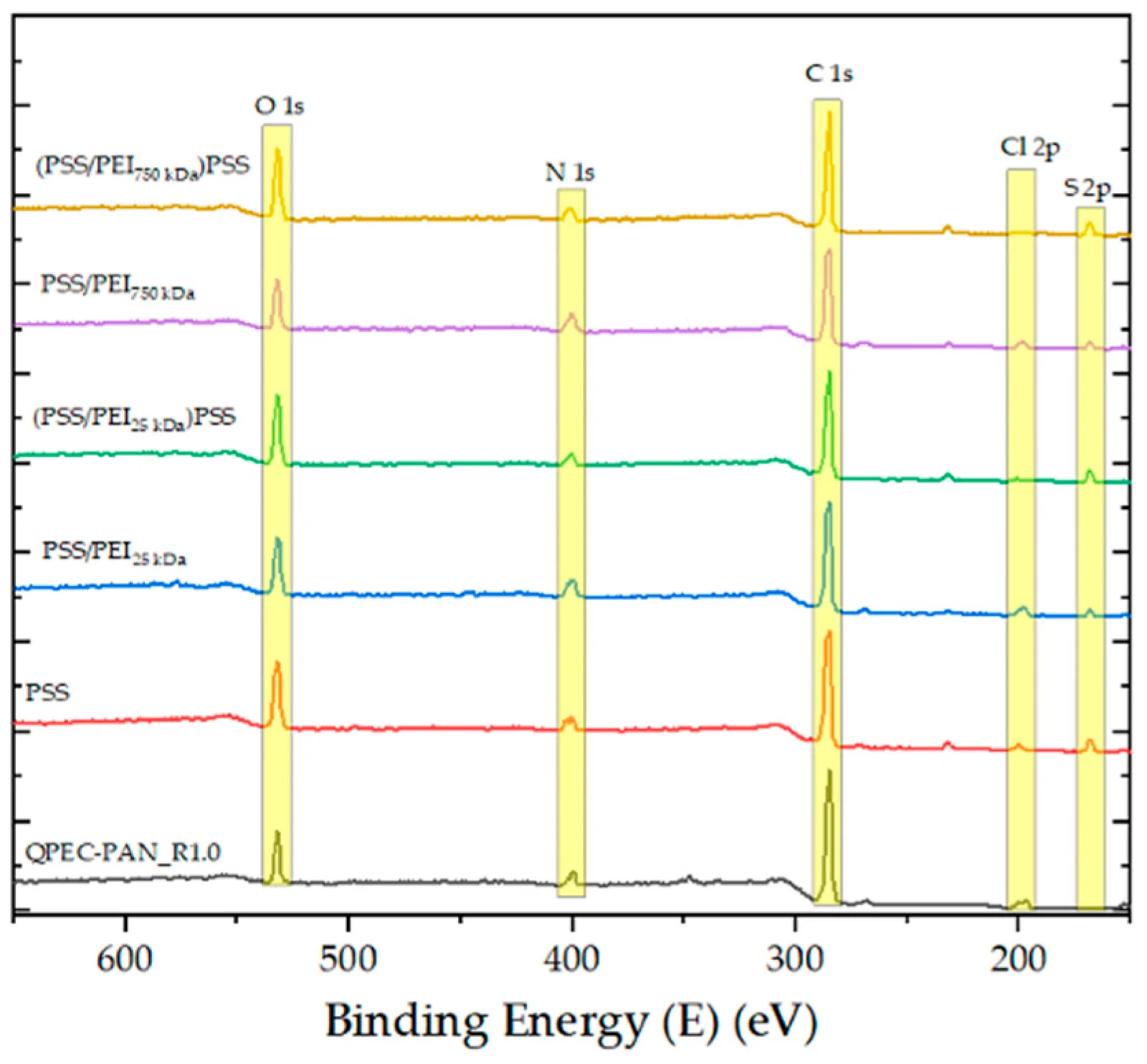
General survey of XPS analysis of the bare and polyelectrolyte-modified AEMs. The performance data for the bare (QPECH-PAN_R 1.0) membrane used as a control group were adapted from our previous study [28] with proper copyright permission from the publisher.

The surface chemical composition of all membranes was examined by XPS (Figure 2 and Table 1). As expected, carbon (C), oxygen (O), nitrogen (N), chlorine (Cl), and sulfur (S) were detected. The appearance of S and the increase in O content after the first PSS layer verify effective deposition of the sulfonated polyelectrolyte. Subsequent PEI deposition increases the N content while decreasing O and S, consistent with successful adsorption of the amine-rich PEI layer. The addition of the top PSS layer again elevates the O and S content.

A clear influence of PEI molecular weight is observed: membranes modified with 750 kDa PEI show a higher N content and higher N/S and N/O ratios compared to those modified with 25 kDa PEI. These trends reflect the hyperbranched architecture and higher amine density of high-molecular-weight PEI, resulting in a thicker or more densely packed interlayer. Additionally, the thickness of the deposited PSS/PEI/PSS multilayers can be comprehensively estimated through the attenuation behavior of the substrate-specific elements in the XPS spectra. Since the maximum probing depth of XPS at a 0° emission angle is approximately 10 nm, the complete disappearance of a substrate signal signifies a coating thickness exceeding this threshold. In our case, the characteristic Cl signal of the PECH backbone remained detectable across all modified membranes, though its intensity systematically decreased with additional layers. This progressive attenuation without complete signal suppression firmly establishes that the LbL modification forms an ultrathin layer with a total thickness well below 10 nm, while successfully shielding the underlying matrix. Furthermore, the remaining Cl atomic concentrations determined in the final membranes provide a reliable basis for a relative comparison of the coating thickness and coverage trends among the different modified samples.

**Figure 3:**
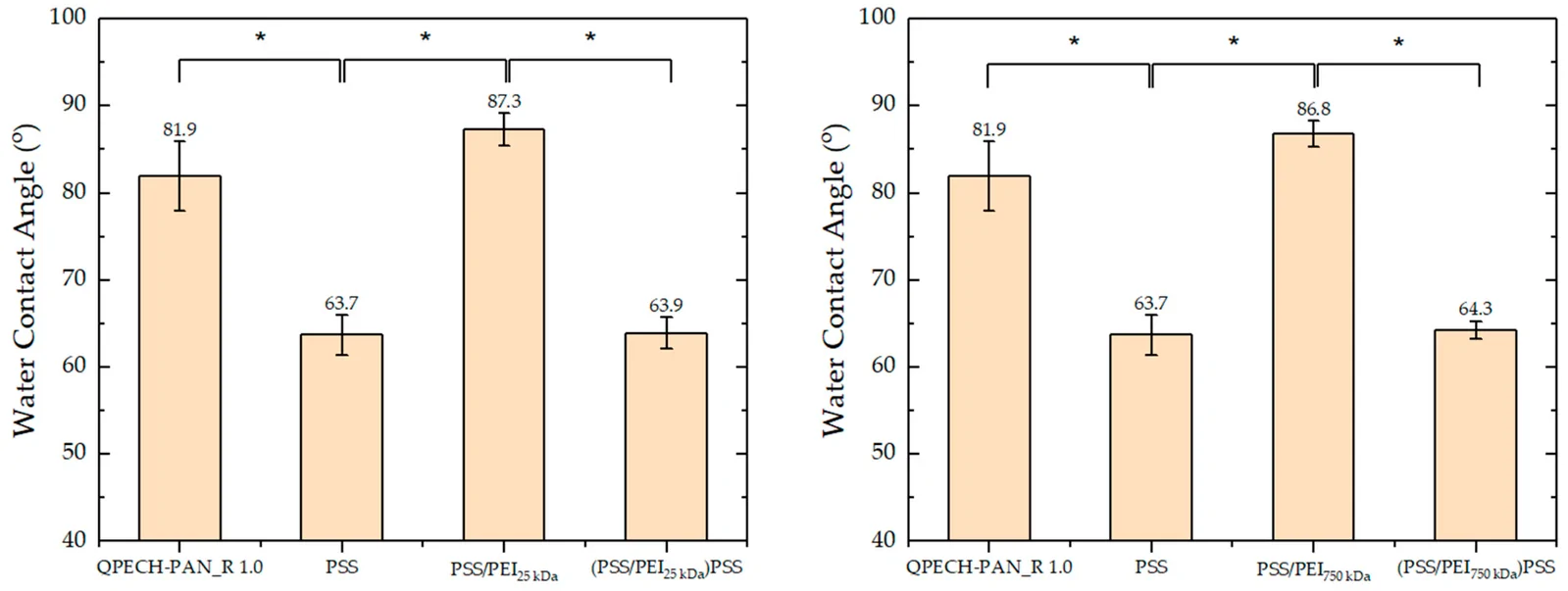
WCA of the bare and polyelectrolyte-modified AEMs (one asterisk (*) denotes that the values significantly differ at the *p* < 0.05 level). The performance data for the bare (QPECH-PAN_R 1.0) membrane used as a control group were adapted from our previous study [28] with proper copyright permission from the publisher.

Figure 3 shows the water contact angle (WCA) results. The pristine AEM exhibits moderate hydrophobicity. After PSS deposition, the WCA drops significantly due to the hydrophilic sulfonate groups. PEI deposition increases the WCA to nearly the original value, reflecting the lower polarity of amine groups. The final PSS top layer again decreases WCA, restoring hydrophilicity. These trends align with the expected polarity characteristics of the respective polyelectrolytes. Similar trends have been observed in the literature, showing that surface characteristics depend on the nature of the modified polyelectrolyte [29,30]. No statistically significant WCA difference is observed between membranes incorporating PEI of different molecular weights, suggesting that the terminating layer predominantly governs hydrophilicity.

**Figure 4:**
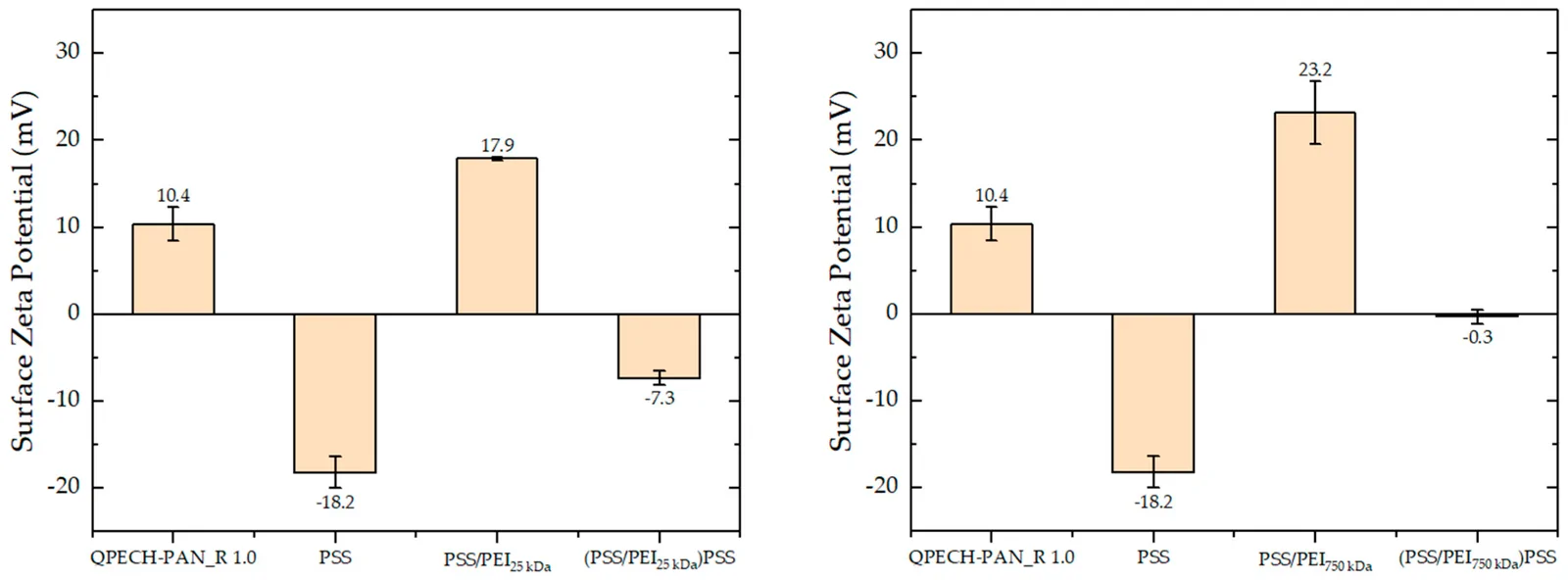
Surface zeta potential of the bare and polyelectrolyte-modified AEMs. All data groups exhibit statistically significant differences within themselves (*p* < 0.05). The performance data for the bare (QPECH-PAN_R 1.0) membrane used as a control group were adapted from our previous study [28] with proper copyright permission from the publisher.

Surface zeta potentials (Figure 4) provide additional insights into the charge characteristics. The pristine membrane exhibits a positive charge due to quaternary and tertiary ammonium groups [28]. After PSS deposition, the charge reverses to negative due to the negatively charged sulfonate groups. Subsequent PEI layers restore a positive charge, with the 750 kDa PEI producing a higher positive potential than the 25 kDa variant, consistent with its higher amine density. This result is consistent with XPS analysis (Table 1). Deposition of the final PSS layer again yields negatively charged surfaces. Notably, the membrane incorporating high-molecular-weight PEI exhibits a surface zeta potential closer to neutrality compared to the other samples, indicating a more balanced surface charge environment. This behavior can be attributed to the increased nitrogen content and denser amine-rich structure of the high-molecular-weight PEI interlayer, which enhances electrostatic interactions between oppositely charged polyelectrolyte layers and promotes partial charge compensation within the multilayer assembly. It should be emphasized that this near-neutral surface charge does not imply the formation of classical zwitterionic structures, which typically require specific functional groups and are associated with well-defined hydration layers. Instead, the observed behavior reflects a charge-balanced interfacial environment resulting from the interplay between the positively charged PEI and negatively charged PSS layers. Such a balanced surface charge may reduce strong electrostatic interactions between the membrane and charged species in solution, thereby influencing ion transport and fouling behavior. In particular, minimizing excessive electrostatic attraction can help suppress the binding of multivalent ions and reduce interfacial resistance, contributing to improved performance under mixed-ion conditions. Similar findings have been reported in the literature, demonstrating that the surface charge is affected by the molecular weight of the modified molecule [20,29]. Together, SEM, XPS, WCA, and zeta potential measurements confirm successful stepwise deposition and reveal that the molecular weight of the PEI interlayer meaningfully affects the surface composition and charge distribution of the final AEMs.

In the LbL deposition method, one of the most significant design parameters is the choice of terminating layer, which is known as the odd/even effect [31]. In this study, the top layer was designed to be negatively charged, as this creates fouling-resistant surfaces due to the more hydrophilic structure of negatively charged polyelectrolytes. Another reason is to exploit electrostatic repulsion between the surface and ions to enhance selectivity for negatively charged divalent ions. In most studies in the literature, the terminating layer was a negatively charged polyelectrolyte layer for the two reasons stated above [29,30,31,32].

### 3.2. RED Performance Tests

RED performance was evaluated using NaCl solutions and mixtures containing divalent ions (Na_2_SO_4_). Table 2 summarizes OCV, power density, and flow-related parameters for monovalent ion conditions. In all cases, increasing flow velocity increases OCV and gross power density. It is important to distinguish the influence of hydrodynamic conditions from intrinsic membrane properties when interpreting RED performance. Increasing flow velocity reduces the thickness of the diffusion boundary layer adjacent to the membrane surface, thereby decreasing non-ohmic resistance and enhancing ion transport. As a result, all membranes exhibit improved OCV and power density at higher flow velocities, consistent with previous studies [23,33,34]. However, hydrodynamic effects alone cannot fully explain the observed differences between membranes. Since all membranes were tested under identical flow conditions, the reduction in boundary layer resistance affects each system similarly. Therefore, the relative differences in performance can be attributed to membrane-specific properties.

At lower flow velocities (0.26 and 0.52 cm/s), the modified AEMs generate slightly lower OCV and power density than the bare AEM. This is expected, as the polyelectrolyte layers increase electrical resistance due to increased thickness and crosslinking density [21,23]. Similar performance penalties for LbL-modified membranes have been reported in RED and ED literature [35,36,37]. Chen’s research group reported that increasing flow velocity resulted in higher OCV and gross power density. Similarly, in their study, the polyelectrolyte-modified AEMs exhibited lower OCV and gross power density compared to the bare AEMs, which was attributed to the additional electrical resistance introduced by the polyelectrolyte layer [23].

However, at higher flow velocity (2.09 cm/s), where boundary layer resistance is minimized, the system approaches a membrane-controlled regime. Under these conditions, the enhanced performance of the (PSS/PEI_750 kDa_)PSS membrane further confirms that the observed improvements are primarily governed by intrinsic and interfacial membrane properties rather than hydrodynamic effects. These findings highlight the importance of evaluating membrane performance under both hydrodynamically limited and membrane-controlled conditions to accurately assess structure-property performance relationships in RED systems.

**Figure 5:**
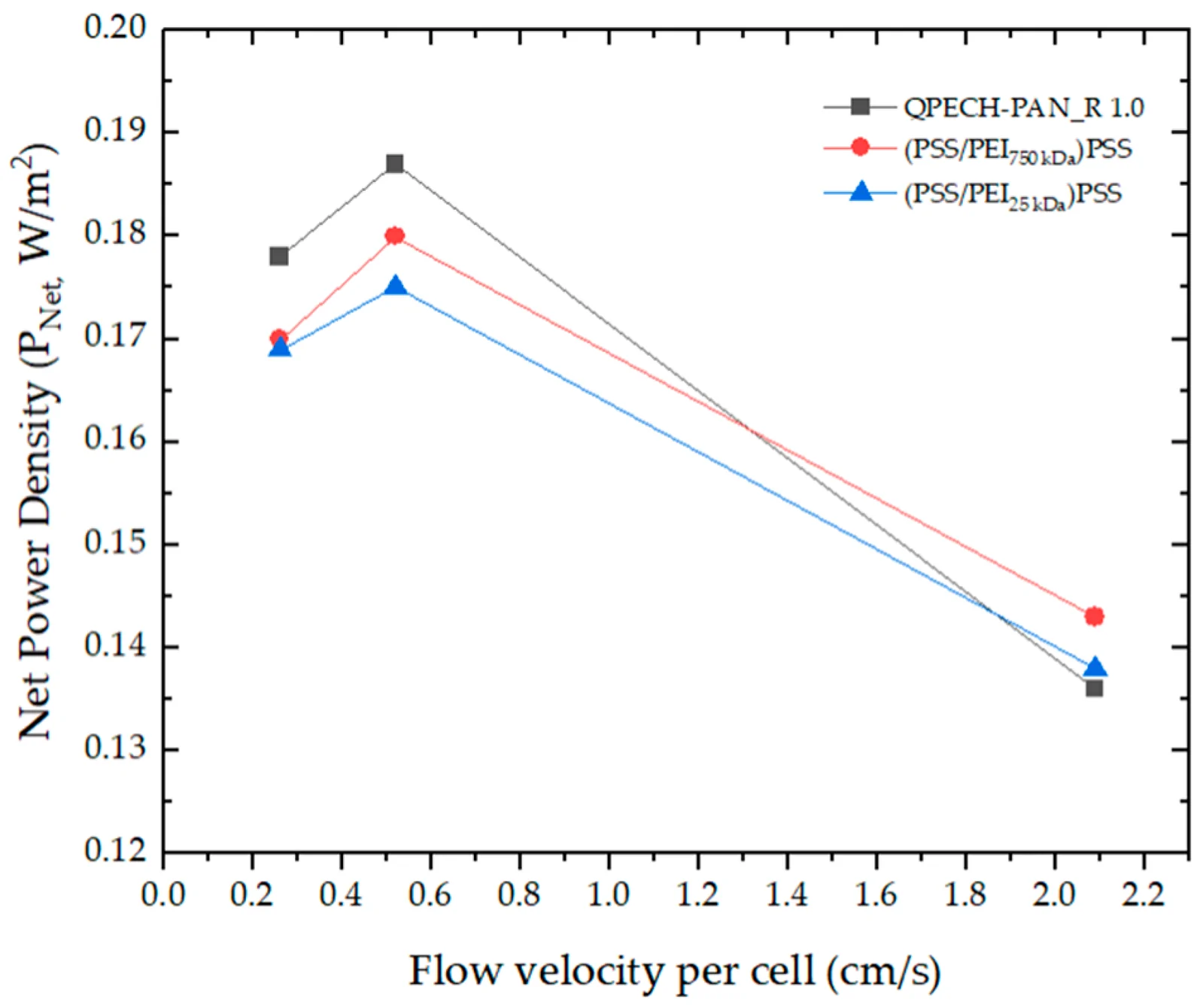
The P_Net_ values of the RED system as a function of flow velocities. The performance data for the bare (QPECH-PAN_R 1.0) membrane used as a control group were adapted from our previous study [28] with proper copyright permission from the publisher.

Figure 5 shows the net power density (P_Net_) as a function of flow velocity. Although gross power density increases with velocity, P_Net_ is maximized at 0.52 cm/s, beyond which increased pumping energy outweighs performance gains. This optimum is consistent with the literature and reflects the balance between reducing mass-transfer limitations and minimizing hydraulic losses [23].

**Figure 6:**
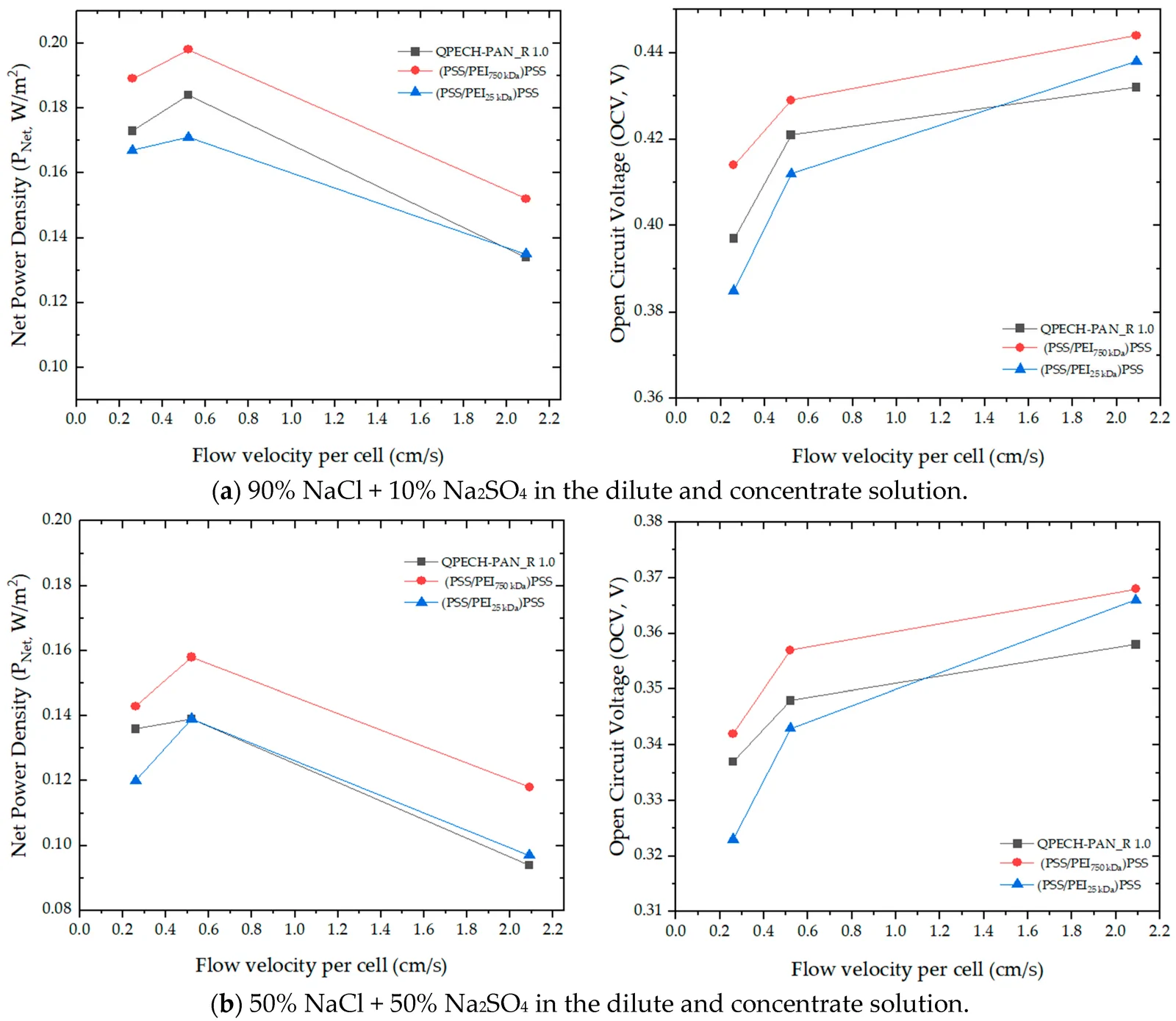
The P_Net_ and OCV values of the RED system as a function of flow velocities. The performance data for the bare (QPECH-PAN_R 1.0) membrane used as a control group were adapted from our previous study [28] with proper copyright permission from the publisher.

To evaluate the effect of divalent ions, RED performance was tested with feed solutions containing 10% and 50% molar ratios of Na_2_SO_4_ (Table 3; Figure 6). It has been reported that natural seawater and river water typically contain a 10% molar fraction of Na_2_SO_4_ [23,38,39]. In this study, this rate (10%) and higher (50%) were investigated to harvest energy from the RED system. Increasing the sulfate fraction decreases both OCV and power density for all membranes. This reduction is expected due to uphill transport of SO_4_^2−^, binding of divalent ions to fixed charge sites, and increased membrane resistance mechanisms, which are widely reported to hinder RED efficiency [23,38,39]. Three uphill ion transport phenomena negatively affect power density: (i) transport of divalent ions against the concentration gradient, (ii) binding of divalent ions to single fixed charge sites, and (iii) binding to multiple fixed charge sites. The first reduces the Nernst potential by requiring counter-transport of monovalent ions, leading to power loss. The second decreases the IEC and permselectivity as the charge sites are occupied by divalent ions, increasing the resistance. The third factor further reduces the free volume, thereby raising electrical resistance and lowering the IEC. Together, these two factors cause significant power loss over time.

The power density values in Table 3 indicate that the interlayer properties have a significant effect on the energy harvesting performance of final AEMs. The (PSS/PEI_25 kDa_)PSS AEM exhibited nearly identical power density to the bare AEM with both divalent ion molar ratios. In contrast, the (PSS/PEI_750 kDa_)PSS AEM achieved the maximum observed power density under both divalent ion concentrations. 

The enhanced Cl^−^/SO_4_^2−^ separation behavior of the (PSS/PEI_750 kDa_ )PSS membrane should be interpreted carefully by considering the actual multilayer architecture. Although direct monovalent selectivity coefficients were not quantified via static methods, the dynamic variations in RED performance parameters under multi-ionic solutions clearly provide a robust, indirect demonstration of the membrane’s preferential selectivity towards Cl^−^/SO_4_^2−^. The improved apparent selectivity is not caused by electrostatic repulsion from PEI, because PEI is positively charged and can interact attractively with anions. Instead, the dominant electrostatic contribution originates from the negatively charged PSS terminating layer. This PSS-rich outer surface creates an interfacial Donnan-type barrier against anion partitioning, which is more pronounced for divalent SO_4_^2−^ than for monovalent Cl^−^ because SO_4_^2−^ experiences a larger electrostatic penalty when entering a negatively charged interfacial region. The molecular weight of PEI influences this process indirectly by controlling the internal structure of the polyelectrolyte multilayer. High-molecular-weight PEI contains a larger number of amine groups and forms a more entangled and compact interlayer through electrostatic complexation with PSS. The correlation between the elemental composition from XPS (Table 1) and the electrokinetic behavior from zeta potential measurements (Figure 4) provides a comprehensive insight into the layer conformation. Specifically, the XPS results showing a significantly higher N content support the formation of a compositionally different and more strongly complexed multilayer. Combined with a more pronounced positive surface potential, these findings firmly confirm that the 750 kDa PEI forms a significantly more compact and densely packed layer compared to lower molecular weight variants. This compact arrangement is attributed to the high chain entanglement and extensive branching characteristic of the 750 kDa PEI macrochains, which effectively crowd the interface with a high density of functional amine groups. This compact interlayer can reduce the effective free volume and increase the tortuosity of the transport pathway, thereby imposing stronger steric and frictional resistance to larger hydrated SO_4_^2−^ ions than to Cl^−^. Therefore, the improved Cl^−^/SO_4_^2−^ transport behavior of the high-molecular-weight PEI system is suggested to be related to the combined effect of the PSS-derived Donnan-type exclusion at the outer interface and the reduced free volume/greater compactness of the PEI_750 kDa_-containing multilayer. This interpretation is consistent with the RED results obtained in mixed NaCl/Na_2_SO_4_ solutions, where the (PSS/PEI_750 kDa_)PSS membrane exhibited the maximum observed power density and enhanced tolerance to sulfate-containing feeds. Compared with state-of-the-art LbL-modified AEMs reported in the literature, the present approach indicates that a notable trend in SO_4_^2−^ tolerance can be initiated using only 1.5 bilayers, which limits additional transport resistance while still providing an effective interfacial barrier against divalent anion transport [18,19,20,22].

To establish a clearer structure, property, and performance relationship, it is important to distinguish between bulk membrane properties and interfacial effects introduced by surface modification. The bare membrane used in this study (QPECH-PAN_R 1.0) has been previously characterized in detail in our earlier work, where it exhibited an ion exchange capacity (IEC) of approximately 1.09 meq g^−1^ and a relatively low electrical resistance of ~4.1 Ω·cm^2^, reflecting its high fixed charge density and favorable ion transport properties [28]. These values indicate that the bare membrane already possesses intrinsically efficient ion conduction characteristics. In the present study, the LbL modification consists of only 1.5 bilayers, forming an ultrathin coating with a thickness on the order of nanometers. Therefore, the bulk structure of the membrane is expected to remain largely unaffected, and the intrinsic properties such as IEC and electrical resistance are not anticipated to change significantly after modification. Instead, the LbL layers primarily alter the interfacial characteristics of membranes. This interpretation is supported by the RED performance results. Under pure NaCl conditions, the modified membranes exhibit slightly lower power densities than the bare membrane, which can be attributed to the additional interfacial resistance introduced by the polyelectrolyte layers. However, in the presence of divalent ions (SO_4_^2−^), the membrane modified with high-molecular-weight PEI demonstrates enhanced performance. This behavior indicates that ion transport is predominantly governed by interfacial phenomena such as ion selectivity, electrostatic interactions, and suppression of uphill transport, rather than by bulk membrane conductivity. Furthermore, XPS and zeta potential analyses reveal that increasing the molecular weight of the PEI interlayer leads to higher nitrogen content and a more balanced surface charge environment. This near-neutral charge distribution likely reduces strong electrostatic interactions with multivalent ions, thereby enhancing apparent monovalent ion selectivity and improving overall RED performance. Overall, these findings suggest that the molecular weight of the PEI interlayer influences membrane performance primarily through modification of surface chemistry and interfacial transport mechanisms, while the intrinsic bulk properties of the base membrane remain largely preserved.

### 3.3. Stability and Organic Fouling Performance of AEMS

**Figure 7:**
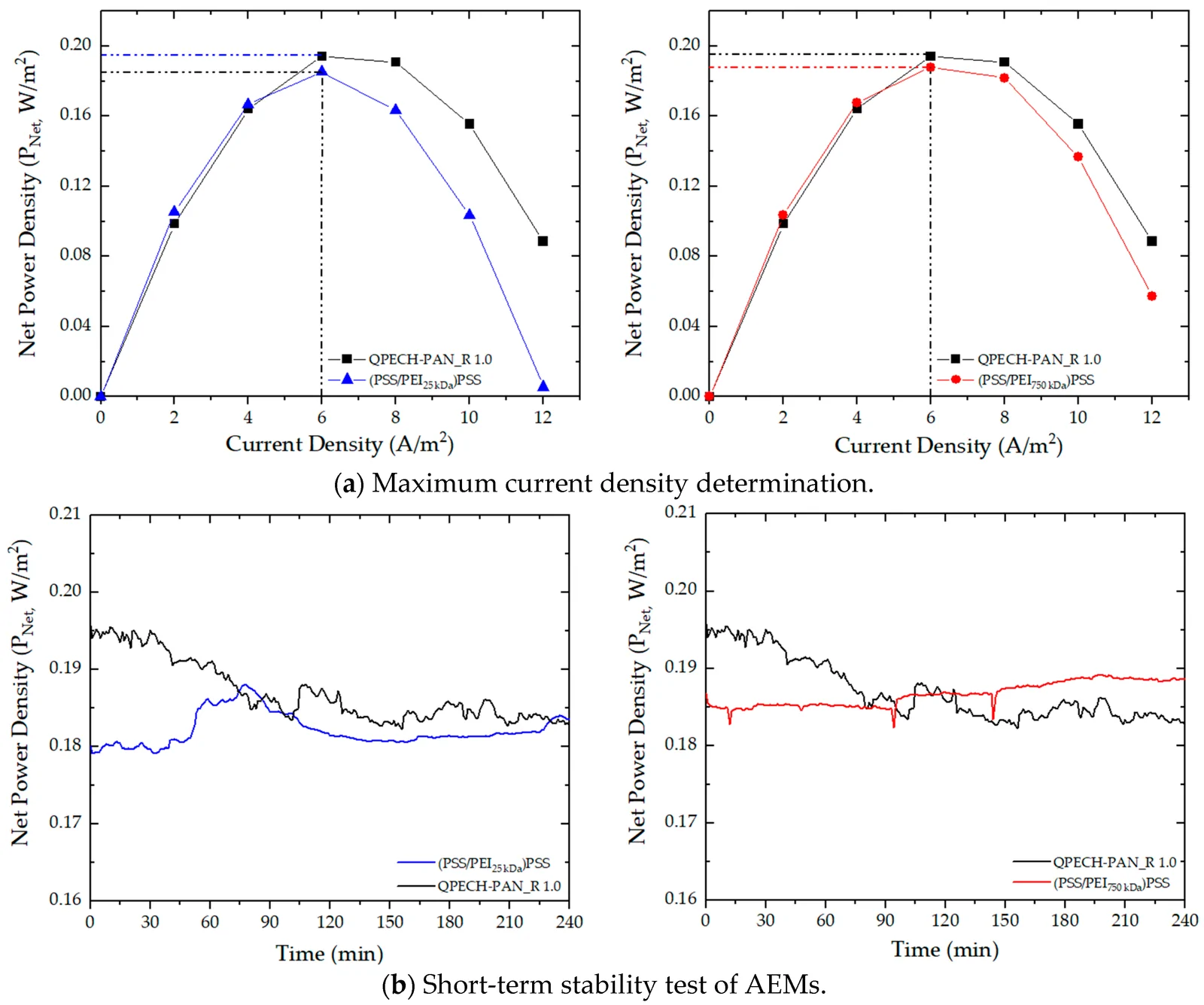
(**a**) Maximum current density determination and (**b**) short-term stability test of the bare and polyelectrolyte-modified AEMs. The performance data for the bare (QPECH-PAN_R 1.0) membrane used as a control group were adapted from our previous study [28] with proper copyright permission from the publisher.

The short-term stability test of AEMs was performed at a constant current density of 6 A/m^2^ and a flow velocity of 0.52 cm/s. Figure 7 illustrates the maximum current density of both bare and polyelectrolyte-modified AEMs. This flow velocity was selected because it produces the maximum observed P_Net_. As shown in Figure 7, the test results indicate that the RED system with bare AEM experienced a P_Net_ loss of approximately 6.8% during operation. However, no loss occurred in the RED system constructed with polyelectrolyte-modified AEMs, and a slight increase in P_Net_ was observed by the end of the test. This power loss in the bare AEM indicates that concentration polarization, which creates non-ohmic resistance at the membrane surface, formed during operation. However, since the surface hydrophilic properties of polyelectrolyte-modified AEMs were much better than those of bare AEM (Figure 3), the formation of a layer that would create resistance on the surface was prevented, and no power loss occurred. Furthermore, the results show that (PSS/PEI_750 kDa_)PSS AEM produced better outcomes, revealing the positive effect of the interlayer with high-molecular-weight of PEI on the short-term performance of the final AEMs.

The short-term stability tests conducted over 4 h provide important preliminary insights into the operational behavior of the membranes under controlled RED conditions. The results indicate that the polyelectrolyte-modified AEMs maintain stable performance throughout the test period, with no observable decline in P_Net_, in contrast to the bare membrane, which exhibits a measurable performance loss. This stability suggests that the LbL layers are structurally robust and remain intact under the hydrodynamic conditions. However, it should be noted that the present stability evaluation is limited to short-term operation and is intended as an initial assessment rather than a demonstration of long-term durability. For practical RED applications, membrane performance must be validated over extended operational periods (days to weeks), under varying feed compositions, and in the presence of real water matrices containing organic matter and multivalent ions. Furthermore, long-term studies should include repeated fouling and cleaning cycles to assess the mechanical and chemical stability of the multilayer structure. Therefore, while the current results confirm the short-term robustness of the LbL-modified membranes, further investigations are required to fully establish their long-term operational stability and scalability. For example, Salvo et al. [40] investigated the long-term operation of a RED system with commercial membranes over 16 days using various real wastewater sources. They identified fouling-induced resistance as the main factor contributing to power loss and introduced an innovative cleaning protocol that allowed continuous system operation. Their findings demonstrated that effective chemical cleaning was crucial for fully recovering the lost power density.

**Figure 8:**
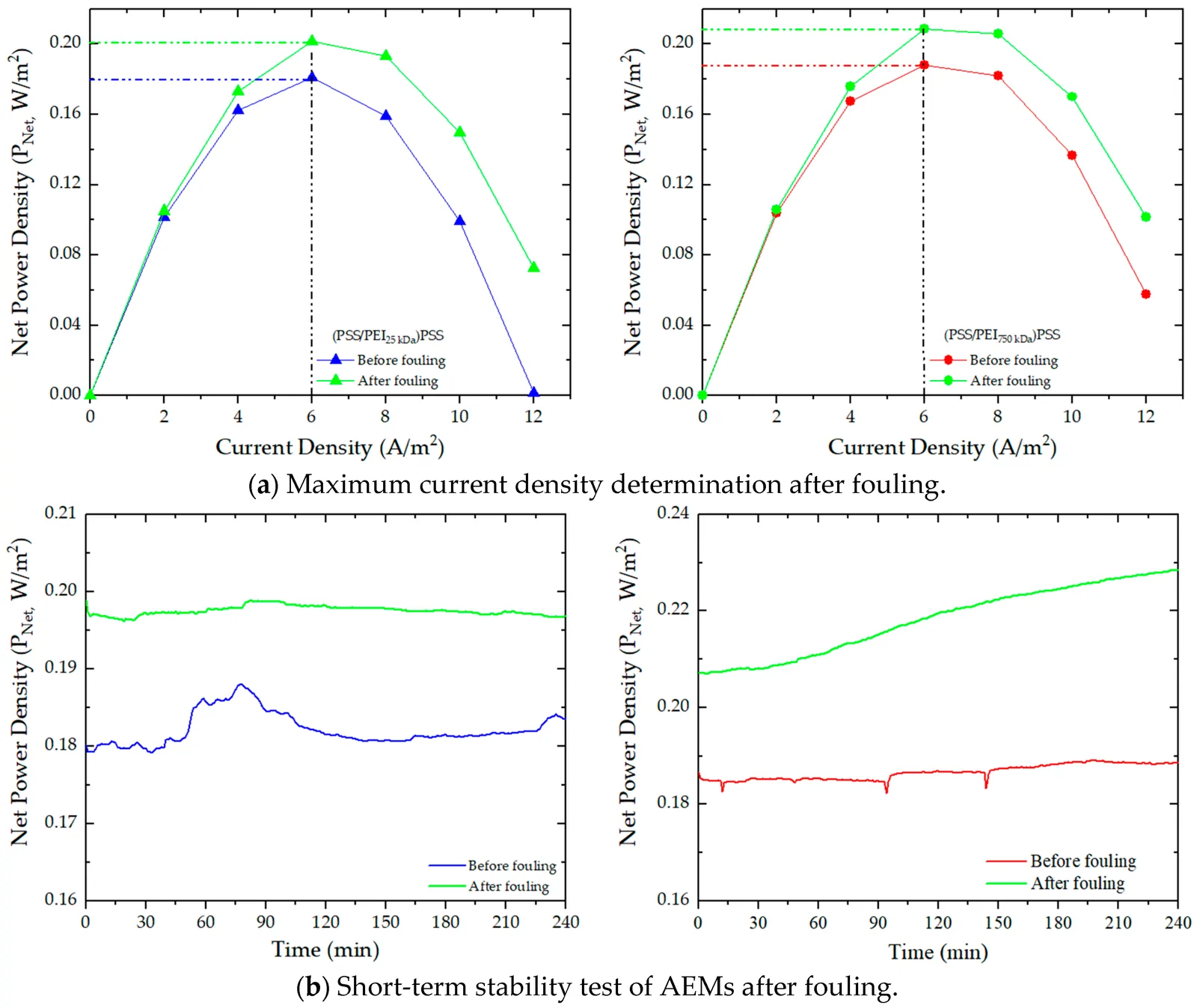
(**a**) Maximum current density determination and (**b**) short-term stability test of the bare and polyelectrolyte-modified AEMs after fouling test.

The determination of fouling behavior of AEMs in the presence of organic matter is crucial for understanding and mitigating performance loss. AEMs are particularly prone to fouling caused by natural organic compounds due to electrostatic interactions between their positively charged surfaces and the negatively charged organic foulants. Consequently, they are more susceptible to this type of fouling than CEMs. Additionally, π–π stacking interactions between aromatic groups in organic pollutants and the crosslinking agents within the AEM further contribute to fouling [41]. These interactions can lead to partially irreversible fouling, depending on the concentration of contaminants. In this study, polyelectrolyte-modified AEMs were statically immersed in a real humic and fulvic acid mixture for 7 d at 25 °C, and their performance was compared with bare and unfouled AEMs. The reason for using a mixture of humic and fulvic acids in our study is that this organic mixture is the major pollutant in river water [17]. The current density and short-term stability test results showed that AEMs with fouling had higher P_Net_ values compared to those without (Figure 8a). This effect is caused by the top coating of the hydrophilic polyelectrolyte PSS, which forms a hydrated surface layer that minimizes the adsorption of organic contaminants, thereby lowering surface resistance. Moreover, the (PSS/PEI_750 kDa_)PSS AEM demonstrated a gradual increase in P_Net_ over time, likely due to the formation of a thin fouling layer that enhanced the membrane’s hydrophilicity and permselectivity. Jucker and Clark investigated the effect of humic and fulvic acid adsorption on the ultrafiltration membrane surface and pores inside [42]. They reported that adsorbed humic and fulvic acid increased the surface hydrophilicity due to the presence of carboxylic acid in their structure.

The observation that polyelectrolyte-modified membranes exhibit improved RED performance after fouling may initially appear counterintuitive, as fouling is generally associated with increased resistance and performance loss. However, this behavior can be explained by considering the nature and structure of the fouling layer formed under the experimental conditions. The humic–fulvic acid mixture used in this study contains functional groups such as carboxylic and phenolic moieties, which can adsorb onto membrane surfaces and increase their hydrophilicity. For the LbL-modified membranes, which already possess a hydrophilic and negatively charged PSS top layer, the adsorption of a thin organic layer may further enhance surface hydration and reduce interfacial resistance. This effect is consistent with previous studies reporting increased hydrophilicity due to the adsorption of natural organic matter [42]. In addition, the fouling layer may act as a secondary selective barrier. The negatively charged functional groups within the adsorbed organic layer can enhance electrostatic repulsion of monovalent anions, while steric hindrance associated with the organic layer may further restrict their transport. This combined effect can suppress uphill transport and improve effective apparent monovalent ion selectivity, leading to increased power density under mixed-ion conditions. In contrast, the bare membrane exhibits a significant decline in performance (around 20% in P_Net_) after fouling. This is attributed to strong electrostatic interactions between the positively charged membrane surface and negatively charged foulants, resulting in the formation of a thicker and more compact fouling layer that increases interfacial resistance. The difference in behavior highlights the importance of surface modification in controlling foulant-membrane interactions.

Similarly, during the 4 h short-term stability tests conducted after fouling, the bare AEM showed an 11.4% decrease in P_Net_, whereas the polyelectrolyte-modified AEMs performed stably throughout the experiment. These results suggest that surface modification with a polyelectrolyte reduces fouling of AEMs, thereby preventing a decrease in P_Net_.

It should be noted that the observed performance enhancement is likely limited to conditions where a thin and loosely structured fouling layer is formed. At higher foulant concentrations or longer operation times, fouling is expected to become detrimental, as widely reported in the literature [42]. Therefore, the present results should be interpreted as a short-term and condition-specific phenomenon, demonstrating the ability of LbL-modified membranes to mitigate fouling effects rather than indicating that fouling is inherently beneficial.

The fouling resistance in this study was evaluated by static immersion in a humic–fulvic acid-containing saline solution, followed by RED performance testing of the fouled membranes. This approach should be interpreted as a controlled preconditioning method to compare the intrinsic affinity of different membrane surfaces toward natural organic matter, rather than as a complete simulation of fouling formation during continuous RED operation. In practical RED systems, fouling is governed not only by membrane-foulant interactions but also by hydrodynamic shear, spacer geometry, boundary-layer development, concentration polarization, and the coexistence of organic matter, colloids, multivalent ions, and inorganic scalants. Therefore, a universal quantitative correlation between static immersion fouling and dynamic RED fouling cannot be assumed. Nevertheless, static adsorption/immersion tests remain useful for identifying relative surface-foulant interaction trends because the early stage of organic fouling is strongly influenced by membrane surface charge, hydrophilicity, and membrane-foulant adhesion. 

In the present work, the statically fouled membranes were subsequently tested under dynamic RED operation with flowing dilute and concentrated streams. Thus, although the fouling layer was formed under static conditions, its effect on energy generation was evaluated under RED-relevant hydrodynamic conditions. The results show that the bare membrane suffered a clear performance loss after fouling, whereas the LbL-modified membranes maintained more stable performance, indicating that the surface modification reduced unfavorable organic foulant interactions. This interpretation is consistent with previous RED fouling studies showing that natural-water fouling is highly complex and cannot be attributed solely to membrane surface fouling. Vermaas et al. [17] reported substantial power-density losses during long-term RED operation with natural waters, where organic matter, colloids, clay minerals, and scaling contributed simultaneously to fouling. Rijnaarts et al. [43] further showed that AEM fouling depends strongly on membrane chemistry, but that AEM performance decline alone explains only a small fraction of total RED power loss, highlighting the importance of spacer fouling and other system-level effects. Therefore, the present static fouling test provides a preliminary but meaningful comparison of membrane-organic matter affinity, while long-term dynamic RED fouling experiments with natural waters are required for full operational validation.

## 4. Conclusions

This study demonstrates that the surface of tailor-made PECH-based AEMs can be effectively modified using electrophoretic LbL deposition of PSS and PEI to enhance performance in RED systems. Comprehensive characterization confirmed that the physicochemical properties of the final AEMs, specifically surface composition, hydrophilicity, and charge distribution, are strongly influenced by the molecular weight of the PEI interlayer. Within the scope of the evaluated parameters, transitioning from a 25 kDa to a 750 kDa PEI interlayer resulted in a clear and consistent enhancement in both apparent selectivity and power density under the investigated conditions. The energy-harvesting experiments revealed that while divalent ions (particularly SO_4_^2−^) decrease performance due to uphill transport and site-binding effects, these losses were substantially mitigated in membranes containing the higher-molecular-weight PEI interlayer. Furthermore, organic fouling experiments using realistic humic–fulvic acid mixtures demonstrated that the hydrophilic PSS top layer markedly improved antifouling behavior, preventing the performance degradation observed in the bare AEM, while short-term stability tests verified that the polyelectrolyte multilayers retained their structural integrity under operating conditions.

To expand upon these findings, further comprehensive investigations utilizing a broader spectrum of intermediate molecular weights would be highly valuable to precisely map the limits of this behavior and fully explore the performance trends across different scales. Additionally, scaling up these electrophoretically modified membranes from laboratory-scale units to larger, pilot-scale stacks represents a critical next step to evaluate their long-term structural durability under continuous hydrodynamic shear, realistic spacer geometries, and repeated chemical cleaning cycles. On a broader scale, the development of such fouling-resistant and selective AEMs carries important environmental and practical implications, representing a significant step toward unlocking the full potential of salinity gradient energy harvesting in realistic aquatic environments. Natural water matrices, such as seawater, brackish water, and industrial effluents, are inevitably contaminated with organic matter and multivalent ions that typically paralyze standard IEMs. By mitigating organic fouling and suppressing power-diminishing transport mechanisms, this interfacial modification strategy offers a viable pathway to maintain steady clean energy outputs, ultimately contributing to the global transition toward sustainable energy symbioses and carbon-neutral power generation from natural salinity gradients.

## Figures and Tables

**Table 1:** XPS analysis of the bare and polyelectrolyte-modified AEMs. The performance data for the bare (QPECH-PAN_R 1.0) membrane used as a control group were adapted from our previous study [28] with proper copyright permission from the publisher.

Membranes	Elements (at.%)					Atomic Ratio		
	C1s	O1s	N1s	Cl2p	S2p	N/S	N/O	S/C
QPECH-PAN_R 1.0	77.6	12.2	6.9	3.4	-	-	0.566	-
PSS	71.0	17.6	6.6	1.5	3.3	2.0	0.375	0.046
PSS/PEI_25 kDa_	69.0	15.4	10.8	2.6	2.2	4.9	0.701	0.032
(PSS/PEI_25 kDa_)PSS	69.2	19.3	6.6	0.9	4.0	1.7	0.342	0.058
PSS/PEI_750 kDa_	69.0	14.7	11.5	2.5	2.3	5.0	0.782	0.033
(PSS/PEI_750 kDa_)PSS	68.4	19.8	7.0	0.5	3.3	2.1	0.354	0.048

**Table 2:** The flow properties, pumping power density, OCV, and generated power density values vary with flow velocity in the presence of monovalent ions. The performance data for the bare (QPECH-PAN_R 1.0) membrane used as a control group were adapted from our previous study [28] with proper copyright permission from the publisher.

Membranes	Flow Velocity (cm/s)	Re	t_res_ (s)	P_Pump_ (W/m^2^)	OCV (V)	Power Density (W/m^2^)
QPECH-PAN_R 1.0 & Fujifilm CEM	0.26	2.30	38.4	0.001	0.415	0.179
	0.52	4.70	19.1	0.004	0.434	0.191
	2.09	18.7	4.80	0.066	0.450	0.202
(PSS/PEI_25 kDa_)PSS & Fujifilm CEM	0.26	2.30	38.4	0.001	0.406	0.170
	0.52	4.70	19.1	0.004	0.424	0.179
	2.09	18.7	4.80	0.066	0.458	0.204
(PSS/PEI_750 kDa_)PSS & Fujifilm CEM	0.26	2.30	38.4	0.001	0.410	0.171
	0.52	4.70	19.1	0.004	0.430	0.184
	2.09	18.7	4.80	0.066	0.460	0.209

**Table 3:** The flow properties, pumping power density, and generated power density values vary with flow velocity in the presence of two different molar ratios of divalent ions. The performance data for the bare (QPECH-PAN_R 1.0) membrane used as a control group were adapted from our previous study [28] with proper copyright permission from the publisher.

Membranes	Flow Velocity (cm/s)	Re	t_res_ (s)	P_Pump_ (W/m^2^)	Power Density (W/m^2^) *	Power Density (W/m^2^) **
QPECH-PAN_R 1.0 & Fujifilm CEM	0.26	2.30	38.4	0.001	0.174	0.137
	0.52	4.70	19.1	0.004	0.188	0.143
	2.09	18.7	4.80	0.066	0.200	0.160
(PSS/PEI_25 kDa_)PSS & Fujifilm CEM	0.26	2.30	38.4	0.001	0.168	0.121
	0.52	4.70	19.1	0.004	0.175	0.143
	2.09	18.7	4.80	0.066	0.201	0.163
(PSS/PEI_750 kDa_)PSS & Fujifilm CEM	0.26	2.30	38.4	0.001	0.190	0.144
	0.52	4.70	19.1	0.004	0.202	0.162
	2.09	18.7	4.80	0.066	0.218	0.184

Note: * 90% NaCl + 10% Na_2_SO_4_ and ** 50% NaCl + 50% Na_2_SO_4_ in the dilute and concentrate solution.

## Data Availability

Data will be made available on request.

## Supplementary Materials

The following supporting information can be downloaded at: https://www.mdpi.com/article/10.3390/polym18172104/s1, Figure S1: A schematic view of the RED system; Table S1: Properties of commercial Fujifilm Type II IEMs used in the RED stack. Refs. [44,45,46,47,48] are cited in Supplementary Materials.

## References

[1] Lee T.D., Ebong A.U. (2017). A review of thin film solar cell technologies and challenges. Renew. Sustain. Energy Rev..

[2] Archer C.L. (2005). Evaluation of global wind power. J. Geophys. Res..

[3] Zarfl C., Lumsdon A.E., Berlekamp J., Tydecks L., Tockner K. (2015). A global boom in hydropower dam construction. Aquat. Sci..

[4] Reguero B.G., Losada I.J., Mendez F.J. (2015). A global wave power resource and its seasonal, interannual and long-term variability. Appl. Energy.

[5] Roberts A., Thomas B., Sewell P., Khan Z., Balmain S., Gillman J. (2016). Current tidal power technologies and their suitability for applications in coastal and marine areas. J. Ocean Eng. Mar. Energy.

[6] Shahbaz M., Rasool G., Ahmed K., Mahalik M.K. (2016). Considering the effect of biomass energy consumption on economic growth: Fresh evidence from BRICS region. Renew. Sustain. Energy Rev..

[7] Moya D., Aldas C., Kaparaju P. (2018). Geothermal energy: Power plant technology and direct heat applications. Renew. Sustain. Energy Rev..

[8] Yip N.Y., Brogioli D., Hamelers H.V.M., Nijmeijer K. (2016). Salinity gradients for sustainable energy: Primer, progress, and prospects. Environ. Sci. Technol..

[9] Logan B.E., Elimelech M. (2012). Membrane-based processes for sustainable power generation using water. Nature.

[10] Ramon G.Z., Feinberg B.J., Hoek E.M.V. (2011). Membrane-based production of salinity gradient power. Energy Environ. Sci..

[11] Weinstein J.N., Leitz F.B. (1976). Electric power from differences in salinity: The dialytic battery. Science.

[12] Veerman J., Saakes M., Metz S.J., Harmsen G.J. (2010). Electrical power from sea and river water by reverse electrodialysis: A first step from the laboratory to a real power plant. Environ. Sci. Technol..

[13] Kuleszo J., Kroeze C., Post J., Fekete B.M. (2010). The potential of blue energy for reducing emissions of CO2 and non-CO2 greenhouse gases. J. Integr. Environ. Sci..

[14] Długołeçki P., Antoine G., Nijmeijer K., Wessling M. (2009). Practical potential of reverse electrodialysis as process for sustainable energy generation. Environ. Sci. Technol..

[15] Tristan C., Fallanza M., Ibanez R., Ortiz I. (2020). Recovery of salinity gradient energy in desalination plants by reverse electrodialysis. Desalination.

[16] Tian H., Wanga Y., Peia Y., Crittenden J.C. (2020). Unique applications and improvements of reverse electrodialysis: A review and outlook. Appl. Energy.

[17] Vermaas D.A., Kunteng D., Saakes M., Nijmeijer K. (2013). Fouling in reverse electrodialysis under natural conditions. Water Res..

[18] Zhao Y., Zhu J., Ding J., Van der Bruggen B., Shen J., Gao C. (2018). Electric-pulse layer-by-layer assembled of anion exchange membrane with enhanced monovalent selectivity. J. Membr. Sci..

[19] Zhao Y., Tang K., Liu H., Van der Bruggen B., Díaz A.S., Shen J., Gao C. (2016). An anion exchange membrane modified by alternate electro-deposition layers with enhanced monovalent selectivity. J. Membr. Sci..

[20] Zhao Y., Gao C., Van der Bruggen B. (2019). Technology-driven layer-by-layer assembly of a membrane for selective separation of monovalent anions and antifouling. Nanoscale.

[21] Güler E., van Baak W., Saakes M., Nijmeijer K. (2014). Monovalent-ion-selective membranes for reverse electrodialysis. J. Membr. Sci..

[22] Gao H., Zhang B., Tong X., Chen Y. (2018). Monovalent-anion selective and antifouling polyelectrolytes multilayer anion exchange membrane for reverse electrodialysis. J. Membr. Sci..

[23] Durmaz E.N., Sahin S., Virga E., de Beer S., de Smet L.C.P.M., de Vos W.M. (2021). Polyelectrolytes as building blocks for next-generation membranes with advanced functionalities. ACS Appl. Polym. Mater..

[24] Tang C., Bruening M.L. (2020). Ion separations with membranes. J. Polym. Sci..

[25] Hong J.G., Chen Y. (2014). Nanocomposite reverse electrodialysis (RED) ion-exchange membranes for salinity gradient power generation. J. Membr. Sci..

[26] Yang S.C., Kim W.S., Choi J., Choi Y.W., Jeong N., Kim H., Nam J.Y., Jeong H., Kim Y.H. (2019). Fabrication of photocured anion exchange membranes using water-soluble siloxane resins as cross-linking agents and their application in reverse electro-dialysis. J. Membr. Sci..

[27] Lee Y.J., Cha M.S., Oh S.G., So S., Kim T.H., Ryoo W.S., Hong Y.T., Lee J.Y. (2019). Reinforced anion exchange membrane based on thermal cross-linking method with outstanding cell performance for reverse electrodialysis. RSC Adv..

[28] Cihanoglu A., Güler E., Kabay N. (2025). The impact of quaternization degree in polyepichlorohydrin-based anion exchange membranes on salinity gradient energy generation by reverse electrodialysis. J. Water Process Eng..

[29] Mulyati S., Takagi R., Fujii A., Ohmukai Y., Matsuyama H. (2013). Simultaneous improvement of the monovalent anion selectivity and antifouling properties of an anion exchange membrane in an electrodialysis process, using polyelectrolyte multilayer deposition. J. Membr. Sci..

[30] Singh K., Sahin S., Gamaethiralalage J.G., Zornitta R.L., de Smet L.C.P.M. (2022). Simultaneous monovalent ion selectivity with polyelectrolyte multilayers and intercalation electrodes in capacitive deionization. Chem. Eng. J..

[31] Grooth J., Oborný R., Potreck J., Nijmeijer K., de Vos W.M. (2015). The role of ionic strength and odd—Even effects on the properties of polyelectrolyte multilayer nanofiltration membranes. J. Membr. Sci..

[32] Ahmad M., Tang C., Yang L., Yaroshchuk A., Bruening M.L. (2019). Layer-by-layer modification of aliphatic polyamide ani-on-exchange membranes to increase Cl−/SO42− selectivity. J. Membr. Sci..

[33] Długołecki P., Ogonowski P., Metz S.J., Saakes M., Nijmeijer K., Wessling M. (2010). On the resistances of membrane, diffusion boundary layer and double layer in ion exchange membrane transport. J. Membr. Sci..

[34] Kim D.K., Duan C., Chen Y.F., Majumdar A. (2010). Power generation from concentration gradient by reverse electrodialysis in ion-selective nanochannels. Microfluid. Nanofluidics.

[35] Lu D., Jin H., Mao Y., Qian Y., Li G., Wang J., Mei Y., Yao Z., Gao Z., Zhang L. (2024). Constructing blocked-nanolayer by surface charge inversion over anion exchange membrane for improved antifouling performance. Desalination.

[36] Chandra A., Bhuvanesh E., Mandal P., Chattopadhyay S. (2018). Surface modification of anion exchange membrane using layer-by-layer polyelectrolytes deposition facilitating monovalent organic acid transport. Colloids Surf. A Physicochem. Eng. Asp..

[37] Lan Y., Huang Y., Qi H., Lai L., Xia L., Zhao Z., Zhao Y. (2022). Alternating electric field-based ionic control and layer-by-layer assembly of anion exchange membranes for enhancing target anion selectivity. Desalination.

[38] Vermaas D.A., Veerman J., Saakes M., Nijmeijer K. (2014). Influence of multivalent ions on renewable energy generation in reverse electrodialysis. Energy Environ. Sci..

[39] Post J.W., Hamelers H.V.M., Buisman C.J.N. (2009). Influence of multivalent ions on power production from mixing salt and fresh water with a reverse electrodialysis system. J. Membr. Sci..

[40] Di Salvo J.L., Cosenza A., Tamburini A., Micale G., Cipollina A. (2018). Long-run operation of a reverse electrodialysis system fed with wastewaters. J. Environ. Manag..

[41] McCloskey B.D., Park H.B., Ju H., Rowe B.W., Miller D.J., Freeman B.D. (2012). A bioinspired fouling-resistant surface modification for water purification membranes. J. Membr. Sci..

[42] Jucker C., Clark M.M. (1994). Adsorption of aquatic humic substances on hydrophobic ultrafiltration membranes. J. Membr. Sci..

[43] Rijnaarts T., Moreno J., Saakes M., de Vos W.M., Nijmeijer K. (2019). Role of anion exchange membrane fouling in reverse electrodialysis using natural feed waters. Colloids Surf. A.

[44] Cihanoğlu A. (2024). Investigation of influencing factors on power generation performance in reverse electrodialysis. Erzincan Univ. J. Sci. Technol..

[45] Jang J., Kang Y., Han J.H., Jang K., Kim C.M., Kim I.S. (2020). Developments and future prospects of reverse electrodialysis for salinity gradient power generation: Influence of ion exchange membranes and electrodes. Desalination.

[46] Altıok E., Kaya T.Z., Güler E., Kabay N., Bryjak M. (2021). Performance of reverse electrodialysis system for salinity gradient energy generation by using a commercial ion exchange membrane pair with homogeneous bulk structure. Water.

[47] Emdadi A., Hestekin J., Greenlee L.F. (2021). Salt screening analysis for reverse electrodialysis. Sustain. Energy Fuels.

[48] Vermaas D.A., Guler E., Saakes M., Nijmeijer K. (2012). Theoretical power density from salinity gradients using reverse electrodialysis. Energy Procedia.

