# Supplementary material for: Tuning Interlayer Molecular Weight in Electrodeposited Anion Exchange Membranes for Enhanced Reverse Electrodialysis Performance
Source: Polymers (Basel). 2026 Aug 29;18(17):2104. doi: 10.3390/polym18172104 (PMC13568115; doi:10.3390/polym18172104)
Supplement: Supplementary file 1 [file polymers-18-02104-s001.zip › polymers-4493067-supplementary.pdf]

SUPPLEMENTARY INFORMATION

Tuning Interlayer Molecular Weight in Electrodeposited Anion Exchange Membranes for Enhanced Reverse Electrodialysis Performance

Aydın Cihanoğlu [1,2]

[1] Department of Chemical Engineering, Ege University, Bornova, 35100 Izmir Türkiye; aydin.cihanoglu@ege.edu.tr; Tel: +90-232-616-7607

[2] Aliağa Vocational School, Refinery and Petrochemical Technology, Ege University, Aliaga, 35800 Izmir, Türkiye

Section S1: Experimental procedures

RED performance tests

The salinity gradient power generation performances of the synthesized AEMs were tested using the RED system (STT Products B.V., BA Schiedam, Netherlands). Figure S1 shows the components and working principle of the RED stack. The electrodes used in the system are Ru-Ir oxide-coated Ti mesh with a 10 cm × 10 cm active area (Magneto Special Anodes BV, BA Schiedam, Netherlands). The polyamide 6-based spacers (Sefar, Switzerland) with 450-micron thickness, 71% porosity, and 38% open areas, and silicon rubber gaskets were used to separate the membranes with a 10 cm x 10 cm active area in the RED system [1]. Commercially available Fujifilm Type II cation exchange membranes (CEMs) were used in our system. The properties of the commercial Fujifilm Type II CEMs shared by the company are given in Table S1. Three AEMs and four CEMs were used in the experiments. Dilute and concentrated solutions were represented as low and high salinity, respectively. The electrode solution was fed as a closed-loop circulation to the electrodes. Two peristaltic pumps adjusted the feeding speed of dilute, concentrate, and electrode solutions in the RED system. The feeding speeds of the solutions were changed during the study, but the speed of the electrode feed solution was constant at 300 mL/min of volumetric flow rate.

The electrode solution (2 L) was prepared using 0.05 M $K_3Fe(CN)_6$, 0.05 M $K_4Fe(CN)_6$, and 0.25 M NaCl ions. To this end, all chemicals were well mixed using deionized water at 25 °C. Then, the mixture was put in a sunlight-proof tank for use. The model salt solutions, 0.017 M as river water and 0.513 M NaCl as seawater, were used as dilute and concentrated solutions, respectively. The main idea of using these concentrations is to mimic the river and seawater conditions because the whole motivation in real applications is to use the RED system where the river and seawater meet. The effect of divalent ions on energy generation performance was also studied in our system. To this end, monovalent (Na^+ and Cl^-) and divalent ions (SO_4^{2-}) were

present simultaneously in a 0.017 M and 0.513 M NaCl solution pair. The Na_2SO_4 concentration was adjusted to be 10% and 50% (molar ratio) of the total solution concentration. Divalent ions were added to both dilute and concentrated solution compartments. The label was coded as 90% NaCl+10% Na_2SO_4 and 50% NaCl+50% Na_2SO_4 for 10% and 50% mole ratios, respectively. All RED tests were carried out at 25 °C. The RED test experiments were repeated four times, and the standard deviations were obtained in the order of 10^{-4}. Since the standard deviation values were too small, they did not add to the values measured. Electrochemical measurements were performed using a Gamry Instrument Reference 3000.

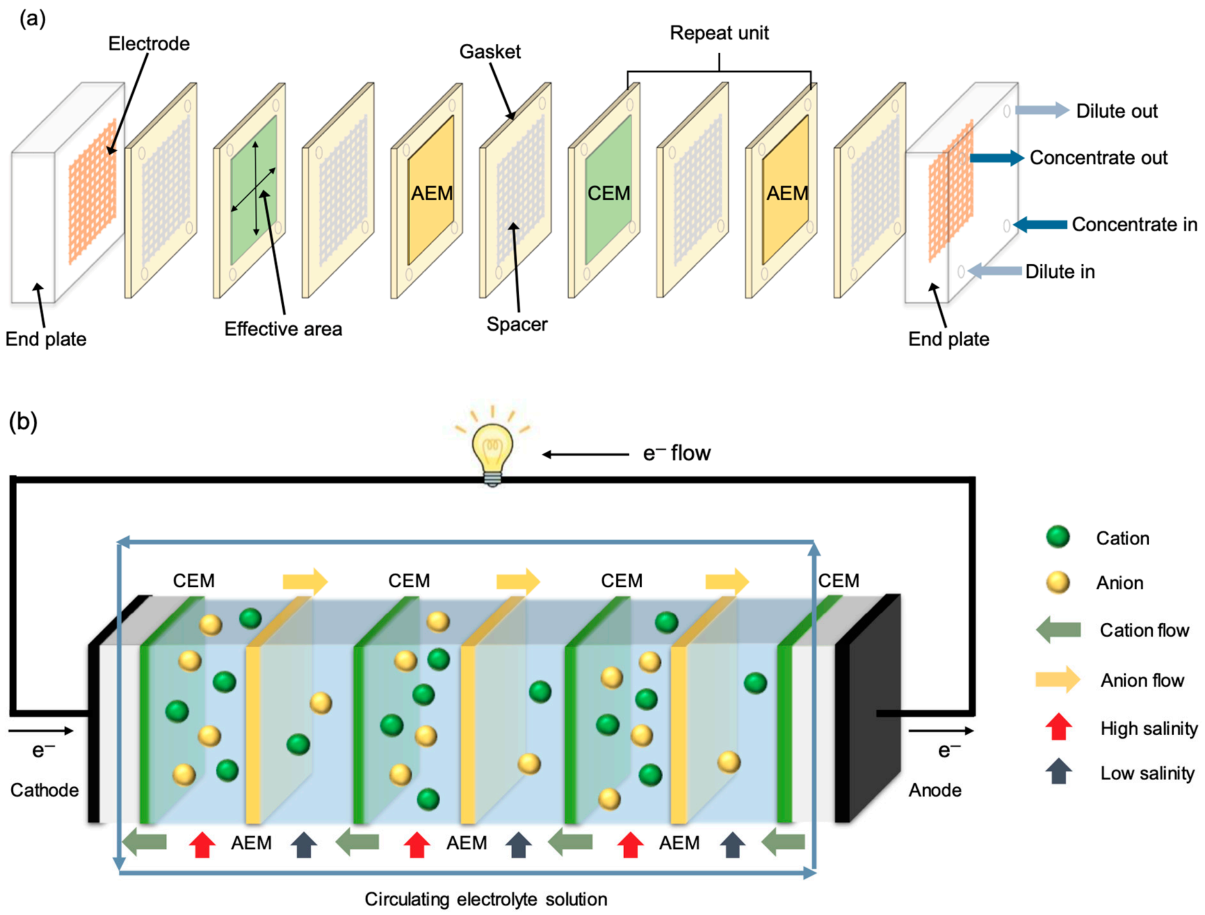

Figure S1. A schematic view of the RED system, a) components, and b) working principle (Reprinted with permission from Yang et al. [2], Copyright 2020, Elsevier).

Table S1. Properties of commercial Fujifilm Type II IEMs used in the RED stack [3].

Properties	CEM
Reinforcement	Polyolefin
Thickness dry (μm)	160
Electrical resistance (Ω cm^2)	8
Permselectivity (%)	96
Ion exchange capacity (meg/g)	1.1
pH stability	4-12
Temperature stability (°C)	40

Section S2: Theoretical background

The difference in salinity between the compartments creates a potential difference known as the Nernst potential. This potential is calculated for each membrane in the presence of monovalent and divalent ions using equations 1 and 2, respectively [4].

$$E_{OCV\ theoric} = \left(\frac{\alpha_{AEM}}{z_-} + \frac{\alpha_{CEM}}{z_+}\right) \frac{RT}{F}\ ln\left(\frac{m_c \gamma_c}{m_d \gamma_d}\right) \tag{S1}$$

$$E_{OCV\ theoric} = \left[\left(\frac{\alpha_{CEM}}{z_{Na^+}}\right) \frac{RT}{F}\ ln\left(\frac{m_c^{Na^+} \gamma_c^{Na^+}}{m_d^{Na^+} \gamma_d^{Na^+}}\right)\right] + \left[(\alpha_{AEM}) \frac{RT}{F} \left[\frac{1}{z_{Cl^-}}\ ln\left(\frac{m_c^{Cl^-} \gamma_c^{Cl^-}}{m_d^{Cl^-} \gamma_d^{Cl^-}}\right) + \frac{1}{z_{SO_4^{2-}}}\ \ln\left(\frac{m_c^{SO_4^{2-}}\ \gamma_c^{SO_4^{2-}}}{m_d^{SO_4^{2-}}\ \gamma_d^{SO_4^{2-}}}\right)^{1/2}\right]\right] \tag{S2}$$

a_{AEM} and a_{CEM} are the membrane permselectivity for AEMs and CEMs, respectively while z_-, and z_+ are the negative and positive ions valences, respectively, R is the universal gas constant (8.31 J/mol K), T is the temperature (K), F is the Faraday constant (96485 C/mol), m is the molality of the solution, g is the activity coefficient of the ion. The subscripts c and d of the constants in the equations represent the concentrated and dilute compartments.

The maximum net power density (P_{Net}) (W/m^2) is calculated as the difference in power densities created by the RED system and consumed by the pumps (P_{Pump}). Equations 3-8 are used for calculating P_{Net} [5].

$$P_{Net} = \frac{(OCV)^2}{4\, R_{Cell}} - P_{Pump} \tag{S3}$$

$$P_{Pump} = \frac{\Sigma \Delta P_i\ \phi_i}{A_{Membrane}} = \frac{\Delta P_c\ \phi_c + \Delta P_d\ \phi_d}{A_{Membrane}} \tag{S4}$$

$$\Delta P = \frac{12\,\mu\,L^2}{\left(\frac{1}{4}\right) d_h^2\, t_{res}} \tag{S5}$$

$$d_h = \frac{4\,\varepsilon}{\left(\frac{2}{h}\right) + (1-\varepsilon)\left(\frac{8}{h}\right)} \tag{S6}$$

$$t_{res} = \frac{L}{U} \tag{S7}$$

$$U = \frac{\phi}{w\,h\,\varepsilon\,N} \tag{S8}$$

R_{Cell} is the total resistance of the RED stack (W cm^2), ΔP is the pressure difference over the feed water compartment (Pa), *F* volumetric flow rate (m^3/s), $A_{Membrane}$ is the total membrane area (m^2), *m* is the viscosity of the solution (Pa s), *L* is the cell length (m), d_h is the hydraulic diameter of the channel (m), t_{res} is the residence time of the solution in the RED stack (s), *e* is the porosity of the spacer, *h* is the intermembrane distance (m), *U* is the linear flow velocity (m/s), *w* is the membrane width (m) and *N* is the membrane number.

The flow regime (Reynolds number, *Re*) of solutions is determined by equation 9.

$$Re = \frac{2\,\phi\,\rho}{w\,\mu\,\varepsilon} \tag{S9}$$

r is the electrolyte solution density (kg/m^3).

The power density loss was calculated using equation 10.

$$Power\ density\ loss\ (PL, \%) = \frac{(P_{Net})_{NaCl} - (P_{Net})_{NaCl+Na_2SO_4}}{(P_{Net})_{NaCl}}\ 100 \tag{S10}$$

References

[1] A. Cihanoğlu, Investigation of influencing factors on power generation performance in reverse electrodialysis, Erzincan Univ. Journal of Science and Technology 17 (2024) 853-869.

[2] J. Jang, Y. Kang, J.H. Han, K. Jang, C.M. Kim, I.S. Kim, Developments and future prospects of reverse electrodialysis for salinity gradient power generation: Influence of ion exchange membranes and electrodes, Desalination 491 (2020) 114540.

[3] E. Altıok, T.Z. Kaya, E. Güler, N. Kabay, M. Bryjak, Performance of reverse electrodialysis system for salinity gradient energy generation by using a commercial ion exchange membrane pair with homogeneous bulk structure, Water 13 (2021) 814.
[4] A. Emdadi, J. Hestekin, L.F. Greenlee, Salt screening analysis for reverse electrodialysis, Sustain. Energy Fuels. 5 (2021) 6135-6144.
[5] D.A. Vermaas, E. Guler, M. Saakes, K. Nijmeijer, Theoretical power density from salinity gradients using reverse electrodialysis, Energy Procedia 20 (2012) 170-184.
